# Recent advances and educational strategies in diagnostic imaging for temporomandibular disorders: a narrative literature review

**DOI:** 10.3389/fneur.2025.1597312

**Published:** 2025-05-26

**Authors:** Ruopeng Zhao, Xin Xiong, Zhenlin Li, Liming Zhang, Haolun Yang, Zheng Ye

**Affiliations:** ^1^Department of Radiology, West China Hospital, Sichuan University, Chengdu, Sichuan, China; ^2^State Key Laboratory of Oral Diseases & National Center for Stomatology & National Clinical Research Center for Oral Diseases, West China Hospital of Stomatology, Sichuan University, Chengdu, Sichuan, China; ^3^Rehabilitation Medicine Center, Department of Rehabilitation Medicine, West China Hospital, Sichuan University, Chengdu, Sichuan, China

**Keywords:** temporomandibular disorders, diagnostic imaging, curriculum development, medical education, artificial intelligence

## Abstract

**Introduction:**

Temporomandibular disorders (TMD) are a group of orofacial conditions characterized by pain and dysfunction of the temporomandibular joint (TMJ) and surrounding musculature. Imaging plays a crucial role in diagnosis and treatment planning. However, educational content on TMD imaging in medical and dental curricula has lagged behind recent technological advances.

**Methods:**

This review analyzes the current status of TMD imaging education based on a synthesis of literature and educational practices. It highlights discrepancies across institutional curricula and evaluates emerging strategies such as interdisciplinary learning, artificial intelligence (AI)-assisted tools, and simulation-based training.

**Results:**

TMD imaging education is found to be inconsistent and underdeveloped globally, with significant variability in curriculum design and limited integration of modern imaging technologies. Current training programs lack standardized guidelines, resulting in knowledge gaps and increased risk of clinical misjudgment. Early findings suggest that AI and simulation tools can enhance educational outcomes.

**Discussion:**

To bridge the gap between clinical practice and technology, a standardized, evidence-based educational framework is essential. Future strategies should include interprofessional collaboration, AI-driven diagnostic support, and immersive simulation environments. Implementing these measures will enable clinicians to accurately interpret TMD imaging and improve patient care.

## 1 Introduction

Temporomandibular disorders (TMD) are a group of conditions characterized by structural, functional, or physiological alterations in the masticatory system, which may also be associated with other systemic or comorbid conditions (1). Globally, the prevalence of TMD varies in the general population. A meta-analysis reported a global prevalence of approximately 34%, with regional variations: 47% in South America, 33% in Asia, 29% in Europe, and 26% in North America (2). The etiology of TMD is multifactorial and includes anatomical factors, trauma, parafunctional habits, psychosocial components, or some rare syndrome (2–5). The development of TMD is influenced by both genetic predisposition and environmental factors. Its chronicity is regulated by various mononucleotides, while emotional disorders, such as depression and anxiety, can exacerbate pain perception and further impair quality of life (6–8). The Diagnostic Criteria for Temporomandibular Disorders (DC/TMD) provide a standardized framework for diagnosing TMD, incorporating both physical assessments and psychosocial evaluations to capture the multifactorial nature of these disorders (9, 10). DC/TMD emphasizes the importance of detailed patient history and clinical examination, particularly for pain-related conditions such as myalgia and arthralgia. Functional evaluations, including polysomnography, are instrumental in diagnosing sleep-related parafunctional activities like bruxism, which can exacerbate TMD symptoms. Devices like the BruxOff provide ambulatory monitoring of masticatory muscle activity during sleep, aiding in the identification of nocturnal bruxism episodes. Furthermore, the integration of advanced 3D articulators, could help the functional analysis of the stomatognathic system. Additionally, screening tools have been developed to assist dental practitioners in the early detection of TMD, enhancing diagnostic accuracy and enabling timely intervention (11, 12). Recognizing that TMD encompass not only joint-related issues but also muscular and functional aspects of the entire stomatognathic system underscores the necessity for a multidisciplinary diagnostic strategy, and no single treatment modality is universally effective (13). Medical imaging provides an objective perspective for assessing the degree of joint involvement and revealing hidden joint surrounding muscles abnormalities, which helps to diagnose and guide treatment.

Traditional two-dimensional imaging techniques, such as panoramic radiography and plain film radiographs, are commonly employed as initial screening tools due to their accessibility and cost-effectiveness, they possess inherent limitations, including superimposition of anatomical structures and reduced sensitivity to early osseous changes. These constraints often necessitate the use of advanced imaging modalities for comprehensive evaluation. In recent years, significant advancements have been made in three-dimensional (3D) TMJ and masticatory muscle imaging. Cone beam computed tomography (CBCT) and magnetic resonance imaging (MRI) have emerged as primary imaging modalities, while ultrasound imaging has demonstrated preliminary potential in this domain (14). For instance, CBCT has emerged as a key method for assessing degenerative changes in the mandibular condyle, with improved sensitivity in detecting surface lesions compared with conventional methods (15). Meanwhile, linkage with artificial intelligence (AI) has also become a current research direction in CBCT (16). Disc displacement is frequently detected on MRI in asymptomatic individuals, and unwarranted intervention may disturb the existing mechanical equilibrium (17). Ultrasonography (US) further enhances diagnostic imaging by quantifying masseter stiffness. Studies have shown that increased elasticity values in patients with TMD are associated with symptom severity, although standardization across protocols remains a challenge (18, 19).

Choosing the appropriate imaging method and interpreting the image with full consideration of the imaging characteristics remains a challenge for the clinician, which requires more advanced and comprehensive training. Systematic reviews highlight the effectiveness of advanced technologies such as virtual reality (VR) and augmented reality (AR) in skill acquisition and objective assessment, while also highlighting the need to integrate these tools with traditional methods to optimize educational outcomes (20). Therefore, there is a need for a structured professional training system to improve clinicians' diagnostic skills.

However, education on TMD-related imaging has not received sufficient attention and there is still a void regarding the development of its related technologies and possible future directions for it related education. This review aims to address this educational gap by systematically evaluating the current status of TMD imaging education, analyzing existing challenges, and exploring future directions.

## 2 Methods

This review was conducted to synthesize and critically evaluate recent advances and educational strategies in diagnostic imaging for TMD.

### 2.1 Search strategy

Relevant literature was identified through searches in PubMed, Embase, and Web of Science. Keywords used included “temporomandibular disorders”, “diagnostic imaging”, “cone-beam computed tomography”, “magnetic resonance imaging”, “ultrasonography”, “education”, and “curriculum development”.

### 2.2 Inclusion and exclusion criteria

Articles were included if they: (1) Focused on imaging modalities (CBCT, MRI, US) related to TMD diagnosis. (2) Discussed educational strategies or curriculum development in dental or medical education concerning TMD.

### 2.3 Study selection

Two reviewers independently screened titles and abstracts for relevance (RZ AND XX). Full-text articles were assessed to ensure they met inclusion criteria. Disagreements were resolved through discussion to reach consensus.

### 2.4 Data extraction and synthesis

Main data extracted included study design, imaging modalities discussed, educational approaches evaluated, challenges identified, and proposed recommendations for educational improvements. Findings were organized thematically into current status, advances in imaging techniques, educational strategies, challenges, and future directions to ensure a comprehensive and structured analysis.

## 3 Current status of TMD imaging education

Formal training in the diagnosis and management of TMD, including imaging analysis, has long been limited within dental and medical education. Currently, no widely accepted TMD curriculum guidelines exist for predoctoral dental education, leading to considerable variability in clinician training across institutions (21). A survey of dental schools in the United States and Canada found that while TMD-related content has been incorporated into some curricula, training in TMD imaging diagnosis and clinical management remains insufficient in certain institutions and, in some cases, includes outdated information (22). The absence of standardized instruction results in inconsistencies in graduates' ability to assess the appropriate indications and clinical applications of TMD imaging. This deficiency may contribute to decreased confidence in clinical decision-making among new practitioners, thereby increasing the likelihood of suboptimal or inappropriate patient care (23).

The severity of this issue is further compounded by the longstanding separation between dental and medical education, commonly referred to as the medical-dental divide (24). Dentistry and medicine have functioned as independent disciplines, creating barriers to the integrated diagnosis and treatment of conditions that span both fields, such as TMD (25). General dentists, primary care physicians, and other healthcare professionals often lack systematic training in TMD management, particularly in critical areas such as TMJ imaging interpretation. Their fragmented cognitive structure and disciplinary isolation further hinder effective diagnosis and treatment. A 2020 report by the National Academy of Sciences, Engineering, and Medicine (NASEM) underscored this educational gap, revealing that a large proportion of medical practitioners exhibit substantial deficiencies in core competencies related to TMD diagnosis and treatment (26). Even in medical schools that offer relevant coursework, there remains a significant disparity between the depth of theoretical instruction and its practical application (27). Abnormal occlusal patterns can lead to uneven loading of the TMJ, predisposing individuals to joint dysfunction, muscle hyperactivity, and subsequent degeneration (28, 29). Several studies have underscored that malocclusion—particularly Class II malocclusion, unilateral crossbite, and anterior open bite—might disrupt mandibular kinematics and increase the risk of disc displacement and myofascial pain (30). Therefore, imaging education should not only emphasize the interpretation of joint morphology but also train students in the radiographic tracing and functional analysis of occlusal discrepancies. Integrating this content will enhance clinicians' ability to correlate occlusal risk factors with imaging findings, improving diagnostic accuracy and enabling targeted interventions (31).

However, discrepancies also exist in the continuing education courses on TMD imaging for practicing clinicians. While some courses adhere to evidence-based medicine principles and offer systematic theoretical and practical training, others rely on anecdotal experience or outdated concepts, lacking a scientific foundation (32). Consequently, clinicians should navigate conflicting perspectives and inconsistent diagnostic and treatment recommendations when enhancing their professional skills. This challenge underscores the need for consensus-driven TMD imaging training programs and standardized clinical practice guidelines.

From an imaging perspective, traditional two-dimensional techniques, such as panoramic radiography, have long been widely used in TMD evaluation; however, they provide limited diagnostic information. CBCT and MRI have enhanced the efficiency of TMD imaging diagnosis (33). Nevertheless, many general dentists have not yet fully mastered the interpretation of CBCT and MRI, partly due to insufficient professional training. In some regions, this knowledge gap has resulted in an over-reliance on referrals and even unnecessary imaging examinations (34). For instance, a recent review highlighted that due to inadequate education and awareness among dental practitioners, CBCT referrals often lack clear clinical indications, leading to unnecessary imaging (35). This not only increases healthcare costs but also exposes patients to avoidable radiation or medical interventions. Conversely, insufficient familiarity with advanced imaging techniques may lead to the underutilization of imaging resources, potentially compromising the accurate diagnosis of TMD and resulting in overlooked pathological changes (36). Therefore, strengthening the training and standardization of TMJ imaging guidelines for dental practitioners is crucial for optimizing imaging-resource allocation, improving diagnostic accuracy, and enhancing patient outcomes.

In summary, TMD imaging education remains unstandardized, with a disconnect between technological advancements and curriculum content, as well as insufficient interdisciplinary training. Before addressing potential solutions, it is essential to first examine the imaging modalities themselves. The following section reviews the primary imaging techniques used in TMD care—CBCT, MRI, and US—emphasizing their clinical applications and educational implications.

## 4 Advances in imaging modalities for TMD

The development of imaging technology has improved the ability to observe the TMJ and its related structures. Different imaging techniques have their own advantages in the diagnosis of TMD. Therefore, mastering the indications and rational application of these techniques is an important part of clinician training. At present, the three most clinically valuable techniques in the imaging evaluation of TMD-related diseases include CBCT, MRI, and US. This section will further discuss the clinical application of various imaging technologies in the diagnosis of TMD, the latest research progress, and their impact on clinical training.

### 4.1 Advances in cone-beam computed tomography

CBCT provides high-resolution imaging of hard tissues through continuous cross-sectional images and three-dimensional reconstruction, while delivering a relatively lower radiation dose than conventional computed tomography (37). It is primarily employed to assess the bony components of the TMJ and is particularly effective in detecting bony changes, including alterations in condylar morphology and joint space narrowing, which are indicative of osteoarthritis or degenerative joint disease (38).

From the perspective of dental education, the use of CBCT presents increased requirements for clinical training. Dental practitioners benefit from standardized training not only in the safe acquisition of CBCT images but also in the interpretation of three-dimensional volumetric data. Additionally, they should develop the ability to differentiate normal anatomical variations from pathological changes with confidence (39). For instance, mild flattening of the condylar surface may represent a normal adaptive response, observed in approximately one-third of asymptomatic adults (40). Therefore, clinical training should focus on enhancing diagnostic accuracy to reduce the likelihood of misdiagnosis.

An essential aspect of CBCT education is adherence to the “as low as reasonably achievable” (ALARA) principle to minimize radiation exposure (41). During training, it is important to emphasize that each CBCT scan should have a well-defined clinical indication and use the smallest possible field of view (FOV) and the lowest radiation dose while maintaining diagnostic accuracy (42). Accordingly, educators should focus on teaching the appropriate indications for TMJ CBCT. Current guidelines suggest that imaging is most appropriate when TMD patients present with red flags or when specific pathological conditions—such as osteoarthritis, fractures, or tumors—are suspected to influence treatment decisions (43). Conversely, routine CBCT is generally not recommended for patients without clear clinical indications. Additionally, thoroughly documenting the clinical rationale for CBCT examinations in patients' medical records is considered a best practice in training (44). This approach could support clinical decision-making, encourage critical thinking, and foster awareness of ethical and forensic considerations.

CBCT training also needs to keep pace with developments in the field. AI-driven CBCT image enhancement technology has increasingly become a research focus. For instance, one study applied a deep learning algorithm to optimize CBCT image quality, thereby improving the detection of TMJ osteoarthritis changes (noise-optimized temporomandibular joint CBCT imaging) (45). Simultaneously, AI has advanced in the automated interpretation of CBCT scans. A 2021 study proposed an AI-based dental CBCT diagnostic system capable of automatically identifying specific pathologies in images (46). As AI technology continues to mature, future dental curricula may incorporate training on AI-assisted diagnosis, emphasizing its clinical applications and the validation methods for its diagnostic recommendations under human supervision.

In summary, CBCT represents a significant advance in TMJ imaging by providing detailed views of bony structures with convenience and moderate cost. Educational programs must cover the proper use of CBCT—from justification and safe operation to interpretation of findings—ensuring that clinicians understand both its power and its limitations.

### 4.2 Advances in magnetic resonance imaging

MRI is regarded as the gold standard for assessing the soft tissue of the TMJ, particularly the disc and retrodiscal tissue (47). Compared to CBCT, MRI offers superior visualization of the relative positions of the intervertebral disc and condyle and is effective in detecting joint effusion, inflammation, or edema in surrounding tissues. In clinical practice, MRI is typically chosen when internal misalignment is suspected, such as disc displacement (which may or may not be accompanied by reduction), or for preoperative evaluation to accurately assess the position of the disc and the overall condition of the joint (48).

MRI findings closely correlate with the clinical staging system and could be used to guide management decisions of TMD. For instance, Wilkes stage III internal derangement (early/mid-stage) may show a displaced disc with reduction and mild bone changes, whereas as the disease progresses to stage V (chronic degenerative stage), MRI may reveal a perforated or severely deformed disc with degenerative bone changes (49). Educators should emphasize these diagnostic frameworks to ensure that clinical dentists not only interpret MRI images but also understand their clinical implications. In addition to evaluating disc position, MRI could detect other pathological changes, such as synovitis (contrast-enhancing joint tissue), joint effusions (fluid within the joint cavity that appears as high signal on T2-weighted images), and early bone changes in the pre-osteoarthritic stage, such as bone marrow edema (50). Modern curricula should focus on these MRI signs and integrate them with clinical findings as part of a comprehensive TMD evaluation. Studies have shown that many asymptomatic individuals exhibit TMJ disc displacement on MRI, and clinical monitoring along with conservative treatment should remain the primary strategies (51). Educators need to emphasize the importance of avoiding overtreatment based solely on imaging findings (52).

Recent advances in TMD-related MRI include the use of high-field magnets (e.g., 3T MRI) to enhance the resolution of TMJ images and the application of new technologies, such as proton density or volume sequences, to more clearly depict thin disks (53). Additionally, the interpretation of MRI may soon benefit from AI. Pilot studies have applied deep learning to TMJ MRI, enabling the accurate and automatic detection of disc displacement (54). As these technologies continue to evolve, educators will integrate them into their curriculum and guide students on using AI output as a supplementary opinion when interpreting MRI.

### 4.3 Advances in ultrasonography

US of the TMJ and muscles is an emerging diagnostic method that has gained widespread attention due to its accessibility, lack of radiation, and ability to provide real-time dynamic imaging (55). Although the US was a routine tool for diagnosing TMD, primarily due to the small size and deep location of the TMJ, coupled with limited image resolution, advances in US technology have prompted a re-evaluation of its utility in TMD diagnosis. Modern high-frequency linear transducers (10–15 MHz or higher) could clearly visualize the mandibular condyle, articular eminence, and even the intervertebral disc in specific locations, offering new methods for diagnosing TMD (56).

From a clinical perspective, US offers a distinct advantage in dynamic assessment, allowing the patient to open and close their mouth while observing the joint. For instance, in cases of reducible anterior disc displacement, the disc may be positioned in front of the condyle when the mouth is closed and move back above the condyle upon mouth opening, indicating disc reduction. In contrast, if the disc fails to reduce, it will remain in front of the condyle throughout mouth opening, a condition associated with closed lock symptoms (57). A recent narrative review highlighted that US is particularly valuable for the early diagnosis of TMJ arthritis and for monitoring TMD progression over time, complementing MRI and CBCT findings (58). Furthermore, US can aid in certain interventions. For example, US guidance can enhance the accuracy of TMJ puncture or injection by visualizing needle position in real time, providing important support for the training of relevant operators (59).

From educational perspective, US training requires hands-on practice to develop proper probe placement and image interpretation skills. Students should first familiarize themselves with the normal ultrasound anatomy of the TMJ before they can identify pathological changes (60). US also reinforces their understanding of anatomy and function. When using US, they could recall the anatomy of the joint and the movement of the disc and condyle, enabling them to effectively integrate theoretical knowledge with dynamic visual demonstrations. As TMJ ultrasound phantoms are not yet widely available, most training is currently conducted on actual patients or volunteers.

At present, US is generally considered a promising adjunct to, rather than a replacement for, MRI or CBCT. As technology advances (such as the use of Doppler ultrasound to detect inflammation through blood flow or the use of higher-frequency probes to enhance resolution), the role of US in the diagnosis of TMD may continue to expand (61). Educators need closely monitor these developments, and future dental curricula may incorporate at least basic training in TMJ ultrasound examinations, enabling general practitioners to understand its indications and limitations while allowing specialists to achieve greater proficiency with this technology.

In summary, ultrasonography represents a recent advance in TMD imaging that aligns well with a trend toward minimally invasive, cost-effective healthcare. While still not mainstream, it provides an excellent example to students of how innovation can expand diagnostic options. Including it in discussions or demonstrations can prepare clinicians to utilize or refer for ultrasound when appropriate, and to remain adaptable as the imaging landscape evolves.

## 5 Educational strategies in TMD imaging

A core principle for improving TMD imaging education is that clinicians need to learn to integrate knowledge across disciplines—combining perspectives from multiple fields, such as dentistry, medicine, radiology, and even physical therapy—to gain a comprehensive understanding of TMD. This requires an interdisciplinary approach to teaching. In recent years, a variety of strategies have been proposed and recommended to enhance the training of students and practitioners in this fields (Table 1).

**Table 1:** Educational strategies for TMD imaging.

**Strategy**	**Description**	**Benefits**	**Implementation considerations**
Simulation-based learning	Recreating the TMJ imaging case using a virtual scene	Provides a risk-free, repeatable practice environment Delivers immediate feedback on learning outcomes	Invest in simulation tools (e.g., software, models) Train faculty to ensure scenarios are clinically relevant
AI-assisted training	Integration of artificial intelligence tools (e.g., diagnostic algorithms, intelligent tutoring systems) into the TMD imaging learning process	Offering personalized feedback and decision support	Require support from reliable AI systems and technical infrastructure Maintain a balance between assisted learning and the cultivation of critical thinking Receive specialized training to interpret AI outputs
Interprofessional education	Collaborative training involving multiple disciplines (dentistry, radiology, medicine, physical therapy, etc.)	Cultivates awareness of interdisciplinary collaboration Enhances overall understanding of TMD management Bridges the medical-dental divide	Require cross-sector coordination Require institutional support
Case-based learning	Using detailed patient cases (including history, imaging, and clinical findings)	Enhances critical thinking and diagnostic reasoning by placing imaging in a clinical context Improves retention by linking theory to real-world scenarios	Incorporate concrete examples to enhance critical thinking and diagnostic reasoning Connect theory to real-world scenarios to improve understanding Encourage active learning and group discussions.
Online and multimedia learning	E-learning modules, videos, and interactive multimedia content covering TMD imaging	Implement self-directed learning with standardized core content delivery Incorporate rich media (e.g., 3D anatomy visuals, quizzes) to enhance understanding	A learning platform and IT support are required. Content need to be updated with the latest evidence Active learner engagement is essential (e.g., quizzes, virtual cases) to prevent passive learning

TMD, temporomandibular disorders; TMJ, temporomandibular joint; AI, artificial intelligence; 3D, three dimensional; IT, information technology.

### 5.1 Interprofessional education

One of the most effective teaching strategies is to bring together learners from various health professions to study TMD collaboratively. A recent program developed a 5-h interactive interprofessional education (IPE) course on TMD for multidisciplinary health professionals, including those from fields such as dentistry, medicine, nursing, and physical therapy (62). This course, based on case scenarios, utilized multimedia learning to address the pathophysiology, evaluation (including imaging techniques), diagnosis, and treatment of TMD. Participants could enhance their theoretical knowledge, and increase their confidence in diagnosing and treating TMD as well. The project results indicated that working in a team format simulated real-world scenarios, where dentists collaborate with clinicians or pain specialists, thereby enriching participants' comprehensive understanding of TMD. Additionally, organizations such as NASEM advocate for the integration of IPE into educational curricula to bridge the medical-dental divide (63).

### 5.2 Case-based and clinical scenario learning

The TMD imaging course goes beyond merely teaching imaging knowledge by incorporating case studies that require students to determine whether imaging is necessary, select the appropriate imaging modality, interpret imaging findings, and formulate treatment plans based on these findings. This teaching approach authentically mirrors the clinical decision-making process (64). For instance, an instructor presented the case of a patient with unilateral TMJ pain and limited mouth opening. Students analyzed the clinical presentation, reviewed an MRI revealing disc displacement without reduction, and determined a suitable treatment plan. Through these exercises, students could learn how to interpret imaging results and gain insight into how imaging influences treatment decisions. Furthermore, they developed critical thinking skills and learned when imaging is truly required. As one evidence-based guideline suggests, imaging should be performed when the diagnosis is uncertain or when initial conservative treatment fails.

### 5.3 Simulation and virtual reality training

The integration of simulation technology in dental education has expanded beyond traditional surgical skills training to encompass various fields, including radiology and TMJ examination. Advances in virtual reality (VR) technology have further facilitated the development of innovative teaching models, enabling learners to conduct and analyze imaging examinations in a virtual environment (65). VR headsets or computer-based programs could generate three-dimensional TMJ models using CBCT or MRI data, allowing students to dynamically visualize anatomical structures and thereby enhance their spatial understanding of imaging results. This immersive learning approach reinforces students' comprehension of imaging anatomy.

In radiology education, software tools could simulate the process of adjusting imaging parameters, helping students intuitively grasp how different MRI sequences influence the visualization of intervertebral disks or how slice thickness affects image resolution in CBCT (66). Additionally, simulation-based teaching incorporates role-playing scenarios, in which students practice explaining imaging examination results to patients. This could strengthen their image interpretation skills, and enhances doctor-patient communication, better preparing them for clinical practice as well.

### 5.4 artificial intelligence and computer-aided education

In recent years, AI has demonstrated value in medical and dental education, particularly in enhancing students' ability to interpret radiological images. For instance, AI can automatically annotate key anatomical structures of the TMJ on MRI, such as condyles, articular disks, and articular tubercles, and provide immediate feedback on students' identification accuracy (67). Additionally, AI-driven case databases allow students to input suspected diagnoses and compare them with established cases, offering targeted guidance when diagnostic discrepancies arise.

Although these technologies are still in the early stages of development, they hold great potential for fostering autonomous learning. Integrating AI into oral medicine education could enhance students' learning experiences and prepare them to adapt more effectively to its widespread use in future clinical practice. Moreover, AI can assist in the diagnosis of TMD by analyzing imaging and clinical data (68). Therefore, training students to interpret AI-generated analyses and critically assess its diagnostic recommendations will become an essential component of future clinical education, ultimately advancing intelligent diagnosis and treatment in oral medicine.

### 5.5 Continuing education and online learning

For clinical dentists, flexible learning is essential for professional development. Many dentists and physicians seek to enhance their proficiency in TMD imaging diagnosis through continuing education (CE) programs, online seminars, or short-term residency training. Effective CE formats include interactive online seminars incorporating case discussions, on-demand learning modules that clinicians can complete at their own pace, and mentorship networks, enabling general dentists to consult maxillofacial pain specialists remotely (69). Particularly notable is the growing use of open-access platforms such as YouTube, where clinicians and students alike frequently access lectures, clinical demonstrations, and expert discussions (70). The COVID-19 pandemic accelerated the mainstream adoption of these models, underscoring their utility in disseminating high-quality educational materials globally (69, 71). However, the quality assurance of such diverse educational offerings remains a critical concern, necessitating the establishment of standardized curricula that align CE content with evidence-based practices and clinical guidelines.

### 5.6 Standardized curriculum development

The dental and medical fields are actively promoting a more systematic integration of TMD and maxillofacial pain into predoctoral education. Currently, some dental schools have established dedicated teaching modules on maxillofacial pain to enhance the educational framework. In the United States, the Commission on Dental Accreditation (CODA) recommends the formal inclusion of TMD-related training in dental curricula (72). If these standards are implemented, all dental graduates will be required to demonstrate competency in diagnosing and managing TMD. Standardized learning objectives may encompass understanding the indications for TMJ imaging, identifying common pathological changes in TMJ imaging, and mastering the interdisciplinary referral process for complex cases.

Although these educational reforms have a clear direction, their implementation faces several institutional challenges, including allocating curriculum time, ensuring faculty competency in TMD education, and providing adequate teaching resources. Notably, the primary goal of these reforms is not only to develop technically proficient clinicians in TMJ imaging interpretation but also to cultivate practitioners with strong clinical reasoning skills who can collaborate effectively across disciplines.

## 6 Challenges in TMD imaging education

While the need for improved education in TMD imaging is clear, several challenges hinder the implementation of comprehensive training programs. Recognizing these challenges is the first step in overcoming them (Table 2).

**Table 2:** Challenges and solutions in TMD imaging education.

**Challenge**	**Description**	**Impact on education**	**Proposed solutions**
Lack of standardized curriculum	No uniform curriculum or guidelines exist for teaching TMD imaging across institutions. Topics may be inconsistently covered or omitted in dental and medical training.	Graduates' competence in TMD imaging varies. TMD signs are difficult to identify. The quality of continuing education courses varies.	Develop consistent curriculum guidelines (e.g., through professional organizations). Include TMD imaging in certification standards and board examinations. Standardize continuing education content to align with current evidence.
Limited faculty expertise	Few educators have expertise in TMD or maxillofacial pain imaging.	Educators may have only basic or outdated content. A lack of specialized teachers reduces students' exposure to advanced imaging technologies.	Provide faculty development workshops and training in TMD imaging. Recruit or partner with orofacial pain specialists and radiologists to teach and mentor. Encourage faculty to engage in continuing education and research in TMD imaging to remain current.
Lack of interprofessional collaboration	TMD imaging education is often siloed by profession (dentistry vs. medicine), with minimal cross-disciplinary learning.	Clinicians inadequately evaluate referrals. Collaboration is lacking.	Implement interprofessional education courses. Develop team-based case studies involving multiple disciplines. Emphasize collaborative practice in coursework and continuing education.
Poor integration with clinical practice	Challenges in integrating TMD imaging training into the clinical workflow. Limited practical experience in linking imaging to clinical practice.	Students may struggle to apply acquired knowledge to real-world cases. Students may face challenges in fully utilizing new imaging methods.	Integrate image interpretation exercises into clinical training. Incorporate clinical case discussions. Provide mentorship to support the application of imaging in clinical practice.
Rapid technological advances	Imaging technology and AI diagnostic tools for TMD are evolving faster than curriculum updates.	Some educational content may be outdated. New graduates may lack familiarity with the latest tools and technologies.	Regularly update the curriculum and course materials. Teach fundamental principles that can be applied to emerging technologies. Offer elective courses or seminars on advanced TMD imaging. Provide up-to-date continuing education for practitioners.
Resource constraints	Lack of access to imaging technology and teaching resources. Limited time allocated for TMD-focused courses.	Students may depend on textbooks or low-quality images. Some courses do not offer a comprehensive TMD imaging experience. Advanced simulations and equipment may be unavailable.	Leverage alternative resources, such as online case libraries and virtual simulations. Seek funding or partnerships to acquire necessary imaging tools. Integrate TMD imaging topics into existing courses to optimize limited course time. Use distance learning to share expertise and examples.

TMD, temporomandibular disorders; AI, artificial intelligence.

### 6.1 Fragmentation and the medical-dental divide

As previously discussed, the medical-dental divide resulted in fragmented TMD research. In dental education, students are typically introduced to TMD within the context of occlusion or prosthodontics, while medical students may learn about TMD only briefly during rheumatology or otolaryngology rotations, or not at all. However, existing curricula often fail to effectively integrate these perspectives and lack cross-disciplinary coordination.

Furthermore, divergent approaches to TMD research within dentistry have further deepened this divide. The ongoing debate between the traditional occlusal-based dental approach and the broader musculoskeletal or behavioral perspectives has intensified over time. The 2020 NASEM report highlighted the lack of standardization in TMD training for both dental and medical professionals as a major barrier to effective diagnosis and treatment (26). To address this issue, it is essential to reform the existing curricula, integrate relevant content, and foster cross-disciplinary collaboration. However, achieving these objectives presents challenges, particularly within the current education system.

### 6.2 Lack of standardized content

Even within dental schools, the depth and scope of TMD imaging education vary. Some courses may dedicate considerable time to the anatomy and interpretation of TMJ imaging, while others may exclude this topic entirely. There is no clear consensus regarding the essential knowledge that dental graduates should possess about TMD imaging. As Klasser et al. (22) noted, the absence of a standardized teaching framework often leads educators to rely on personal experience or interests, resulting in inconsistent educational content. Consequently, the development of a consensus curriculum—possibly through collaboration among professional organizations such as the American Association of Dental Education or the International Association for Dental Research—faces numerous challenges, particularly in ensuring agreement among experts on core competencies and the timely incorporation of new evidence.

### 6.3 Faculty expertise and resources

Teaching subjects such as MRI interpretation often requires specialized knowledge that not all dental faculty possess. Many dental schools lack oral and maxillofacial radiologists or maxillofacial pain specialists, making it difficult to systematically teach these topics. Additionally, obtaining imaging materials for instruction is challenging. For example, providing students with MRI cases of the TMJ for review may necessitate collaboration with the hospital's radiology department. Some institutions may also lack CBCT equipment due to cost constraints, and the implementation of VR technology is similarly expensive (73). As a result, although there may be a willingness to teach TMD imaging, practical resource limitations often pose a significant barrier to achieving this educational goal.

Furthermore, faculty members may require ongoing training to stay abreast of the latest developments in imaging technology. Faculty trained prior to the widespread adoption of CBCT technology may need additional training to master it before they can teach it effectively (74). Thus, encouraging faculty professional development in this area is essential; however, this need is often overlooked, with teaching resources typically focused on traditional subjects.

### 6.4 Keeping pace with technological change

TMD-related imaging and management is advancing rapidly, necessitating constant updates to educational content. It is crucial for educators to stay current with the material they teach. A syllabus updated a decade ago may not include ultrasonography or AI and may fail to address the critical guidance of CBCT imaging techniques, which have gained increasing prominence over the past 10–15 years (75). Ensuring that course content remains up-to-date presents an ongoing challenge that requires regular reviews of the latest literature and periodic revisions of course materials, efforts that demand institutional support. Moreover, there may be resistance to curriculum updates. Some faculty members may question the necessity of teaching new techniques, such as TMJ US, particularly if they do not extensively use these techniques in their clinical practice and are unaware that clinical practice patterns are evolving (76).

### 6.5 Overemphasis on imaging versus clinical skills

Educational objectives should not foster the perception that every case of TMD requires imaging. Rather, as repeatedly emphasized, imaging should be employed cautiously and interpreted within the context of the specific clinical situation. Over-reliance on imaging modalities may lead to the neglect of clinical fundamentals (77). While imaging is a valuable supplement to the physical examination, it should never replace it. A practical approach to addressing this issue is to teach evidence-based guidelines that clearly outline the indications for imaging (78). For instance, students can learn that “if the patient has significantly improved after conservative management, imaging may not be necessary,” whereas “if persistent severe functional impairment or unusual symptoms are present, imaging should be considered.”

### 6.6 Interpreting imaging in a biopsychosocial framework

The biopsychosocial (BPS) model, emphasized in modern pain education, underscores the multidimensional nature of pain, wherein biological factors (such as imaging findings) represent only one component of the overall disease mechanism, while psychological and social factors also play a crucial role (79, 80). As a paradigmatic condition within the BPS framework, TMD is closely associated with stress, mood disorders, and other systemic pain syndromes. Therefore, a key challenge in education is guiding students to interpret imaging findings within a broader clinical context. This necessitates not only the integration of imaging interpretation but also the inclusion of fundamental concepts in pain psychology and chronic pain management. As MRI may reveal moderate TMJ osteoarthritis, the patient's pain experience may be primarily influenced by central sensitization or parafunctional habits (81). To address this, educators should present case-based learning that connects imaging findings with cognitive behavioral therapy for pain management or physical therapy for associated muscular issues. Although holistic teaching approaches are gaining recognition, they may conflict conceptually with the traditional biomedical-centered training model. Addressing these challenges requires coordinated efforts at multiple levels, including curriculum planning, standardized accreditation guidelines, and faculty development programs.

## 7 Future directions of education

Looking ahead, several developments and initiatives promise to transform TMD imaging education. Embracing these future directions can ensure that clinicians are equipped with the knowledge and skills to provide excellent, patient-centered care. Some key areas of focus include:

### 7.1 Standardization of curriculum and guideline

Establishing clear and standardized learning objectives for TMD and TMJ imaging has become a central priority in contemporary dental education. Dental schools need to incorporate TMD-related content into their curricula, covering the basic principles, indications, and clinical applications of imaging technologies. This will encourage schools to allocate teaching time efficiently and ensure the systematic and comprehensive nature of TMD imaging education (72). Furthermore, the development and dissemination of standardized teaching resources—such as textbooks, online modules, and clinical case libraries—will help reduce variability in training content across institutions and enhance the quality and consistency of instruction (82). Interdisciplinary collaboration is also crucial for optimizing TMD imaging education. Guidelines developed jointly by dental and medical societies can provide all healthcare providers with unified standards for TMD diagnosis and imaging evaluation, fostering collaboration and information exchange across specialties (83). The implementation of these standardized guidelines will further streamline the TMD imaging evaluation system, allowing it to play a more prominent role in professional examinations and certification assessments. This will ensure that future clinicians are equipped to accurately interpret TMD imaging data and contribute to the standardized advancement of clinical diagnosis and treatment practices. The establishment of a TMD-treatment specialty would not only elevate educational standards but also drive innovation and practices in TMD management, ultimately benefiting both clinicians and patients through improved diagnostic accuracy and treatment outcomes.

### 7.2 enhanced interdisciplinary training and clinics

The emergence of joint training programs, such as mini-residency training or refresher courses, will provide a common learning platform for dentists, pain specialists, radiologists, and physical therapists, enhancing trainees' comprehensive understanding of TMD (84). Dentists will gain insight into how physical therapists manage myofascial pain, while physical therapists will have the opportunity to learn how dentists interpret CBCT images of the TMJ. This interdisciplinary training model will enable future clinicians to better understand each other's professional perspectives.

The establishment of joint programs will also strengthen clinical cooperation and interdisciplinary collaboration. In clinical practice, dentists will be able to identify suspected cases of TMJ autoimmune arthritis on imaging and refer patients to rheumatologists, while rheumatologists will be able to assess whether a patient's earache requires a dental evaluation related to TMD diagnosis and management (85). By fostering interdisciplinary referral awareness, more efficient coordinated care will be promoted, improving patient outcomes and the overall quality of care.

### 7.3 Integration of AI in training and practice

In the future, AI will assist clinicians in learning and providing clinical decision support. In the field of TMD imaging, AI could automatically identify potential erosions or disc displacements in images. Therefore, training programs could integrate AI literacy into the curriculum, teaching students the fundamental principles, advantages, and limitations of AI algorithms. A systematic review of AI applications in TMJ diseases has highlighted the growing academic interest in tasks such as using machine learning to classify TMJ osteoarthritis and predict TMD risk based on comprehensive data (86). Consequently, educators should prioritize helping trainees adapt to this emerging technology, emphasizing the use of AI as a tool to support, rather than replace, clinical judgment. Future clinicians should be able to verify AI outputs and make appropriate adjustments when AI may provide inaccurate results.

### 7.4 Expansion of simulation and virtual training platforms

Simulation technology is advancing rapidly. Simulators may incorporate tactile feedback devices that replicate resistance to jaw movement or joint sounds, allowing trainees to experience and diagnose these conditions (87). Additionally, VR enables students to build a three-dimensional TMJ model to observe the movement of the intervertebral disc or simulate an ultrasound examination, providing virtual feedback to assess image quality. As the cost of these technologies decreases, more schools will be likely to adopt them. Furthermore, online virtual patient cases can be created, allowing students worldwide to manage simulated TMD patients by logging into the system, which offers an automatic tutoring system to support the interpretation of pathology and imaging data (88). The continued development of distance learning also opens up more opportunities for international educational collaboration. This cross-border interaction and knowledge exchange will contribute to the unification and advancement of global educational standards.

### 7.5 Research and evidence-based educational methods

As clinical practice should be evidence-based, teaching methods can also benefit from empirical research. Studies could investigate whether students learn to interpret TMJ imaging more effectively through spaced repetition of cases or whether integrating patient narratives with imaging (while maintaining clinical relevance) enhances student competence. Feedback from recent graduates could help identify which TMD educational content is most and least valuable in clinical practice, and the effectiveness of the education itself should be evaluated. For instance, tracking whether enhanced training improves patient care (e.g., whether clinicians who receive advanced TMD training order imaging studies more appropriately and achieve higher patient satisfaction) could provide valuable insights (89). Such data would offer a strong foundation for educational policymakers.

### 7.6 Patient-centered training and proper use of imaging

In line with the trend toward patient-centered care, future training may include real-life patient interactions to enhance real-life communication skills (90). Specifically in the area of imaging, it is critical to teach students how to explain imaging results to patients in a way that is easy to understand. Therefore, it is necessary to develop tools such as 3D models or animated videos that students can use to practice explaining imaging results to patients rather than simply describing the report (91). In addition, the principle of “treating the patient, not the image” must always be upheld, and imaging technology should be used as an auxiliary means to combine diagnosis and treatment with clinical judgment.

## 8 Limitations and future perspectives

### 8.1 Limitations

Despite providing a comprehensive overview of current imaging modalities and educational strategies for TMD, this review is subject to several limitations.

Firstly, as a non-systematic review, this study is inherently subject to selection bias (92). Articles were selected based on subjective judgment rather than a predefined protocol, which limits reproducibility and increases the risk of overlooking contradictory evidence.

Secondly, the focus of the review remains imaging-centric and educator-centric. Perspectives from patients regarding imaging burden, radiation anxiety, accessibility, and informed decision-making are minimally discussed (93).

Thirdly, while AI is presented as a future enabler, significant risks including algorithmic bias, data security concerns, overreliance on automation, and medico-legal accountability issues were underexplored (94).

### 8.2 Future perspectives of TMD imaging

The future of TMD imaging holds promise, but several unresolved issues need to be acknowledged.

Firstly, without internationally accepted diagnostic imaging protocols for TMD, variability across studies and clinical practices will continue to impair comparability and meta-analytic synthesis. Future efforts must prioritize consensus-based frameworks endorsed by interdisciplinary bodies.

Secondly, imaging modalities risk being misused to “find something to treat” in asymptomatic patients, leading to overdiagnosis and overtreatment (95). A stronger emphasis on clinical correlation and restraint must be embedded into future clinician training.

Thirdly, current imaging studies mostly focus on static diagnosis rather than dynamic disease progression. Future imaging research should prioritize longitudinal cohort studies to track TMD evolution over time and evaluate imaging biomarkers predictive of outcomes (8).

Lastly, future imaging research should integrate patient-reported outcome measures into evaluation frameworks to ensure that technological advances translate into perceptible patient benefits, not just better image quality (96).

## 9 Conclusion

TMD imaging education is crucial for optimizing the use of imaging techniques, such as CBCT, MRI, and US, in diagnosing and treating TMD. It ensures that clinicians can select the appropriate imaging modalities, accurately interpret results, and tailor treatment plans while considering the biopsychosocial aspects of TMD. Contemporary education needs to emphasize interdisciplinary collaboration, evidence-based practice, and a standardized curriculum.
